# Impact of emergency department length of stay before icu admission on patient outcome

**DOI:** 10.1186/2197-425X-3-S1-A151

**Published:** 2015-10-01

**Authors:** R Garcia Gigorro, M Talayero-Giménez de Azcárate, I Sáez-de la Fuente, S Chacón-Alves, Z Molina-Collado, N Lázaro-Martín, J Á Sánchez Izquierdo-Riera, JC Montejo-González

**Affiliations:** Hospital 12 de Octubre, Intensive Care, Madrid, Spain

## Introduction

For Emergency Department (ED) patients the timing of transfer to the Intensive Care Unit (ICU) to receive the most appropriate treatments and early life-sustaining therapies may be an important determinant of outcome.

## Objectives

To analyse the relationship between the ED length of stay and the clinical course of patients once admitted to ICU.

## Methods

An ambispective cohort study of adult patients consecutively admitted to an ICU exclusively from the ED, from October 2011 to March 2013.

Variables recorded were: sex, age, comorbidities, ED length of stay, diagnosis, procedures, complications, severity scores (SOFA and APACHE II), ICU and hospital length of stay and evolution. For the assessment of clinical deterioration a Delta-SOFA score was calculated as the difference between SOFA score at ICU admission and SOFA score on ED admission. Statistical analysis was made using T-test, Mann-Whitney test, Chi-squared or Fisher's exact test as appropriate. The coefficient of Spearman Rank was used to measure correlation between quantitative variables. Data were analysed with STATA v10.0. All *P-*value less than 0.05 were considered significant.

## Results

269 patients were included, 58.7% were male with a median age of 54 years (IQR: 42.5 to 65.5). Median ED length of stay before transfer to ICU for the study population was 277 minutes (IQR: 129 to 622).

**Figure 1:**
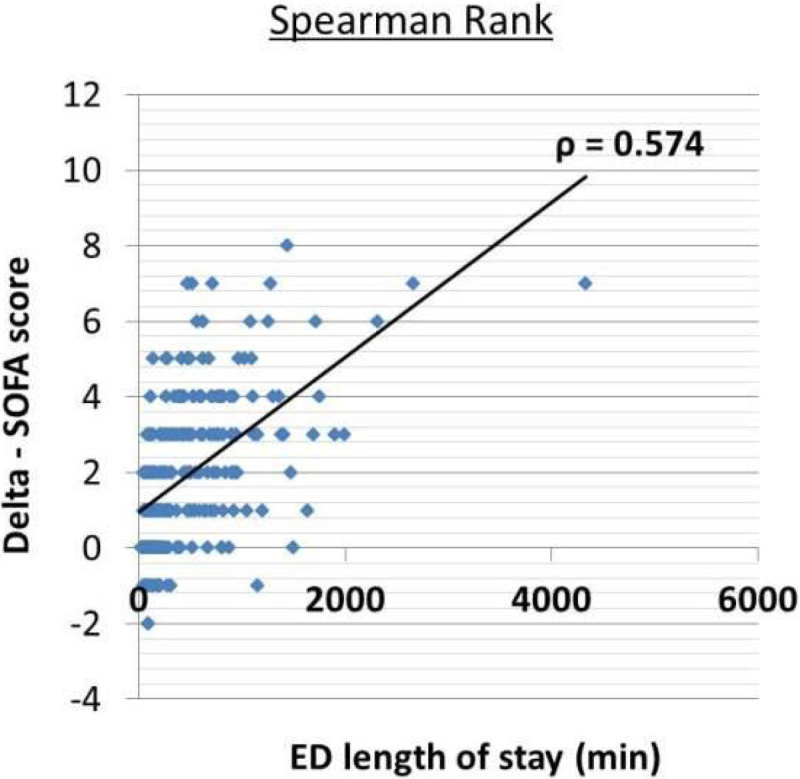
**Correlation Delta-Sofa score an ED time.**

A moderate correlation between ED length of stay and Delta-SOFA score was observed (*r* = 0.57, *P* < 0.001); (Figure 1).

**Figure 2:**
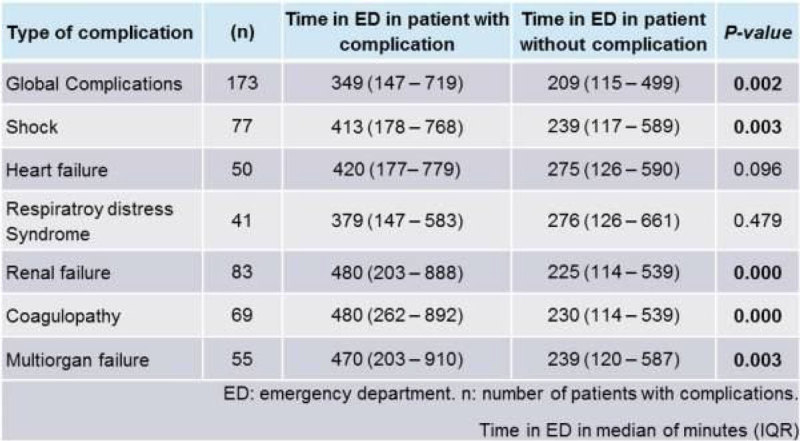
**3 ED time and ICU complications.**

The group of patients who developed ICU complications had a longer length of ED stay compared to those without complications (349 vs. 209 minutes, *P* = 0.002). Particularly, shock, renal failure, haematological complications and multiorgan failure were time-dependent complications (Figure 2). Patients who died in the ICU had a longer length of ED stay (421 vs. 266 minutes, *P* = 0.000).

**Figure 3:**
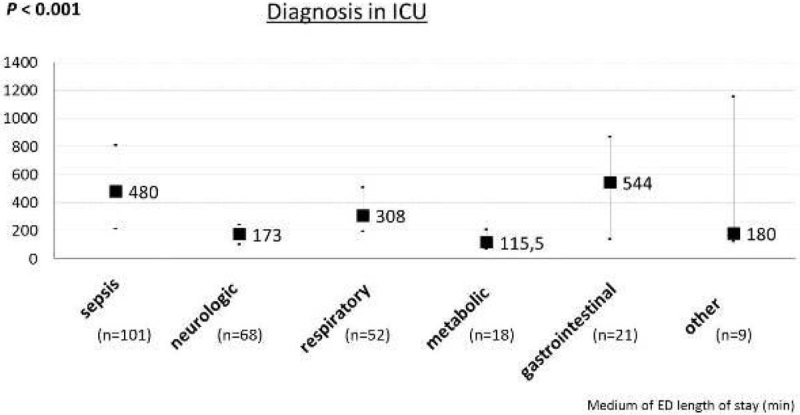
**ED time and diagnosis in ICU admission.**

The ICU diagnosis was associated with a higher ED length of stay (*P* = 0.001); briefly, patients with gastrointestinal or sepsis diagnosis were the most delayed in the ICU admission, (Figure 3). Neither the day (weekday vs weekend) of admission into ICU nor the shift work (day vs night) were associated with ED length of stay; weekday 276 minutes vs. weekend 288 minutes,

*P* = 0.593; or day-time 265 min vs. night-time 395 min, *P* = 0.136, respectively.

## Conclusions

The length of ED stay before the ICU admission is significantly related to worsened outcome, including the development of “time-dependent” complications and increasing the mortality. Only the diagnostic category was associated with the length of ED stay.

